# Genomic Survey of Heat Shock Proteins in *Liriodendron chinense* Provides Insight into Evolution, Characterization, and Functional Diversities

**DOI:** 10.3390/ijms232315051

**Published:** 2022-11-30

**Authors:** Yongchao Ke, Mingyue Xu, Delight Hwarari, Jinhui Chen, Liming Yang

**Affiliations:** 1 College of Biology and the Environment, Nanjing Forestry University, Nanjing 210037, China; 2 Key Laboratory of Forest Genetics and Biotechnology of Ministry of Education of China, Co-Innovation Center for Sustainable Forestry in Southern China, Nanjing Forestry University, Nanjing 210037, China

**Keywords:** *Liriodendron chinense*, heat shock proteins, evolution, structure characteristics, expression pattern

## Abstract

Heat shock proteins (HSPs*)* are conserved molecular chaperones whose main role is to facilitate the regulation of plant growth and stress responses. The HSP gene family has been characterized in most plants and elucidated as generally stress-induced, essential for their cytoprotective roles in cells. However, the *HSP* gene family has not yet been analyzed in the *Liriodendron chinense* genome. In current study, 60 *HSP* genes were identified in the *L. chinense* genome, including 7 *LchiHSP90s*, 23 *LchiHSP70s*, and 30 *LchiHSP20s*. We investigated the phylogenetic relationships, gene structure and arrangement, gene duplication events, cis-acting elements, 3D-protein structures, protein–protein interaction networks, and temperature stress responses in the identified *L. chinense* HSP genes. The results of the comparative phylogenetic analysis of HSP families in 32 plant species showed that LchiHSPs are closely related to the *Cinnamomum kanehirae* HSP gene family. Duplication events analysis showed seven segmental and six tandem duplication events that occurred in the *LchiHSP* gene family, which we speculated to have played an important role in the *LchiHSP* gene expansion and evolution. Furthermore, the Ka/Ks analysis indicated that these genes underwent a purifying selection. Analysis in the promoter region evidenced that the promoter region *LchiHSPs* carry many stress-responsive and hormone-related cis-elements. Investigations in the gene expression patterns of the *LchiHSPs* using transcriptome data and the qRT-PCR technique indicated that most *LchiHSPs* were responsive to cold and heat stress. In total, our results provide new insights into understanding the *LchiHSP* gene family function and their regulatory mechanisms in response to abiotic stresses.

## 1. Introduction

Plant growth and development is to a greater extend affected by various abiotic stresses, including cold, heat, salt, and drought stress [1,2,3]. Nonetheless, plants have evolved complex molecular mechanisms using a wide range of specialized genes and other transcription factors to adapt to prevailing adverse environmental factors [4,5]. These include the heat shock proteins (HSPs), a large family of molecular chaperones [6], highly conserved and widely distributed in prokaryotes and eukaryotes. Research has shown the HSP gene family to play important roles in regulating plant growth and enhancing abiotic stress resistance [7,8]. Under normal conditions, the HSP content has been estimated to constitute about 5% of the total cell protein, and synthesized in large quantities under environmental stress as a response mechanism especially under high-temperature stress, amounting up to 15% of the total cell protein [9]. In detail, the heat shock proteins participate in the folding and unfolding of polypeptide chains, assembly, transport, and the degradation of proteins [10]. Biochemical research on HSPs has shown a classification of five families based on the molecular weight, that is: HSP20, HSP60/GroEL, HSP70/DnaK, HSP90, and HSP100/ClpB [6]. Among them, the HSP20, HSP70, and HSP90 protein families are highly abundant molecular chaperones in the cytoplasm of eukaryotic cells [11,12].

Particularly, the HSP90 class encompasses the heat shock proteins that are highly conserved and widely distributed in the cytoplasm, chloroplast, mitochondria, and endoplasmic reticulum, accounting for 1–2% of total cellular proteins. It exists mainly as a homodimer, containing a 12 kDa N-terminal domain of the ATP-binding site, the 35 kDa intermediate domain, and a 25 kDa C-terminal substrate-binding domain [13,14]. The HSP70 class is one of the most widely distributed and studied heat shock proteins, comprising of a 44 kDa N-terminal ATP binding domain (NBD), 15 kDa substrate binding domain, and about 10 kDa C-terminal domain [15,16]. It is also found that HSP70 family is highly homologous to HSP110 family, showing similarity in gene sequence and structural characteristics. Thus, the HSP110 can be classified as the HSP70 family, even though its molecular weight is relatively large.

The HSP20 is defined by small heat shock proteins, with a conserved structure and molecular weight of 15–42 kDa. They are also ATP-independent chaperone proteins that can bind and isolate the target proteins through conformational changes. Furthermore, HSP20, as the first line of defense against protein misfolding, can prevent protein misfolding and irreversible aggregation. It consists of a variable N-terminal region, a conservative C-terminal region, and a C-terminal extension region. The C-terminal contains an amino acid α-Crystallin domain (ACD domain), mainly composed of a β-Sandwich structure. In particular, HSP20 is the most thermally reactive in plants, because they have an obvious induction effect [17,18].

The HSP gene family has been identified and analyzed in many plants including: *Arabidopsis thaliana* [19], *Oryza sativa* [20], *Populus trichocarpa* [21], and *Zea mays* [22]. Accumulating research has shown that the HSP gene family plays an important role during plant development and abiotic stress resistance. In *A. thaliana, AtHSP17.6A* increased the gene expression, water-loss retention, and seed development during a heat treatment which in turn enhanced the tolerance to salt and drought stress [23]. The overexpression of the *OsHSP16.9* gene in rice increased the salt and drought resistance [24]. *CaHSP70-2* from pepper overexpressed in *Arabidopsis* induced the expression of stress-responsive genes, improving the heat tolerance in *Arabidopsis* [25]. Three genes, *NtHSP90-4*, *NtHSP90-5*, and *NtHSP90-9,* were up-regulated in response to drought, salt, cold, and heat stress in the abscisic acid (ABA)-dependent pathway [14]. The simultaneous induction of *StHSP17.8* and *StWRKY1* following a pathogen invasion in potato elevated the plant defense mechanism [26]. It was also found that the resistance genotypes of sunflower to powdery mildew expressed more HSP70 and defense-related proteins than in sensitive genotypes [27].

HSPs also actively participate in the growth and development of plants. The overexpression of the corresponding GhHSP24.7 homologous gene in *Arabidopsis* and tomato promoted a seed germination [28]. Two genes AtHSP70-16 and AtVDAC3 have been shown to promote an ABA efflux from endosperm to embryo under low-temperature conditions, thereby inhibiting seed germination [29]. The AtHSP90-mediated AtPIN1 distribution regulates auxin distribution, promoting a development in Arabidopsis [30].

The *Liriodendron chinense* (Hemsl.) Sarg. is a deciduous tree in *Magnoliaceae*, mainly distributed in a subtropical climate zone in central China and the south of the Yangtze River basin [31,32]. *L. chinense* is widely cultivated in China for its important high-quality wood and ornamental value. Completion in the genome sequencing of *L. chinense* has provided a basis for genetic characteristics analyses [33]. However, members of the HSP gene family in *L. chinense* have not yet been identified, nor have their functions during abiotic stresses. Therefore, the analyses of the *HSP* genes in *L. chinense* will help expand the geographical distribution and agronomic cultivation of the *L. chinense.* In this research, 7 *HSP90*, 23 *HSP70*, and 30 *HSP20* genes were identified in the *L. chinense* genome. Comprehensive analyses were carried out in the physiochemical properties, phylogenetic relationships, gene structure, conservative motifs, gene replication events, cis-acting elements, 3D protein structures, and protein–protein interactions. In addition, RNA sequencing (RNA-seq) and quantitative real-time PCR (qRT-PCR) were used to analyze the expression patterns of *LchiHSPs* under heat and cold stress. This study will provide a firm base for further experimentations, a theoretical reference, and the comprehension of the biological functions of *LchiHSPs* in abiotic stresses.

## 2. Results

### 2.1. Identification of HSP Gene Members in L. chinense

The *Arabidopsis* HSP protein sequence were used to query the *L*. *chinense* HSP members using the BLASTP and hidden Markov model (HMM) methods. Additionally, the preliminarily identified members were further characterized in the Pfam, SMART, and NCBI databases to determine the final HSP members of *L*. *chinense*. After removing redundant sequences, 60 *LchiHsp* genes were identified, which included: 7 HSP90s, 23 HSP70s, and 30 HSP20s. The identified *LchiHSPs* were renamed according to the chromosomal position as: *LchiHSP90-1~7, LchiHSP70-1~23*, and *LchiHSP20-1~30* (Figure S1). Among them, the LchiHSP90 family was the smallest containing an amino acid range of 325 (LchiHSP90-5) ~ 816 amino acids (aas.) (LchiHSP90-1). The predicted molecular weight (MW) ranged from approximately 36.14 (LchiHSP90-5) to 93.26 kDa (LchiHSP90-1), and the isoelectric point (PI) values were between approximately 4.46 (LchiHSP90-5) and 5.06 (LchiHSP90-7). The lengths of the LchiHSP70 proteins ranged from 514 (LchiHSP70-4) to 884 aas. (LchiHSP70-7), with a molecular weight of 56.63 (LchiHSP70-4) ~ 98.26 kDa (LchiHSP70-7) and isoelectric point (PI) values between approximately 4.74 (LchiHSP70-16) and 8.9 (LchiHSP70-10). The LchiHSP20 family had the largest protein sequence lengths, ranging from approximately 137 (LchiHSP20-14 and LchiHSP20-28) to 340 aas. (LchiHSP20-26). The predicted molecular weight (MW) was approximately from 15.57 (LchiHSP20-14) to 38.71 kDa (LchiHSP20-26) and isoelectric point (PI) values ranged between approximately 4.96 (LchiHSP20-21) and 9.57 (LchiHSP20-25). The subcellular localization showed that 50% of the LchiHSPs were mainly localized in the cytoplasm, 20% in the chloroplast, and 10% in the mitochondria. Additional information on the LchiHSPs is listed in Table 1.

### 2.2. Phylogenetic Relationship of the LchiHSP Genes in L. chinense

**Figure 1:**
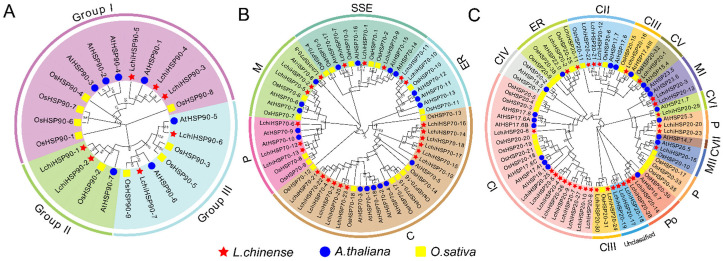
Phylogenetic analysis of HSP90 (**A**), HSP70 (**B**), and HSP20 (**C**) proteins from *L. chinense*, *Arabidopsis*, and rice. The phylogenetic tree was constructed using MEGA-X based on the neighbor-joining method with 1000 bootstrap replicates. The subfamilies are distinguished by different color schemes. Genes from *L. chinense* (Lchi), *Arabidopsis thaliana* (At), and *Oryza sativa* (Os) are marked by red star, blue circle, and yellow rectangle in the inner circle, respectively. C: cytoplasm, CI–CVII: cytoplasm I–VII, ER: endoplasmic reticulum, MI–MII: mitochondria I–II, P: plastid, and Po: peroxide.

To better understand the evolution and the phylogenetic relationship of the HSP genes in *L. chinense*, *A. thaliana*, and *O. sativa*, we constructed a rooted phylogenetic tree using the full protein sequences (Figure 1, Table S1). For better presentation purposes, we classified the HSP protein sequences according to previous classification methods. LchiHSP90 was divided into three subfamilies, of which group I contained three members (LchiHSP90-3, -4, -5), two in group II (LchiHSP90-1 and LchiHSP90-2), and group III (LchiHSP90-6 and LchiHSP90-7) (Figure 1A). Twenty-three HSP70 sequences were classified into five groups (C, P, M, SSE, and ER). Group ER and group C had the least members (2 proteins) and the most members (12 proteins), respectively. Interestingly, group P, M, and SSE had the same number of proteins, three proteins, (Figure 1B). Thirty LchiHSP20s were classified into 12 subfamilies based on the phylogeny and subcellular localization analyses. In detail, 10 genes localized in the cytoplasm (CIs), were classified as: 3 CIIs, 3 CIIIs, 0 CIVs, 1 CV, 1 CVI, and 0 CVII; 2 mitochondria (MIs) as 1 MIIs; 2 in the peroxisomes (Pos); 2 in the endoplasmic reticulum (ER); and 2 in the plastids (Ps). Surprisingly, three *L. chinense* HSP*20* genes *(*HSP*20-17, -18,* and *-19*) did not cluster into any subfamily (Figure 1C). In total, we concluded that most LchiHSP20s (18/28) belonged to CI-CVII, except three unclassified LchiHSP20s. These findings indicated that the cytoplasm might be the main functional region of the LchiHSP20s.

### 2.3. Evolution of HSP Genes from Algae to Angiosperms

**Figure 2:**
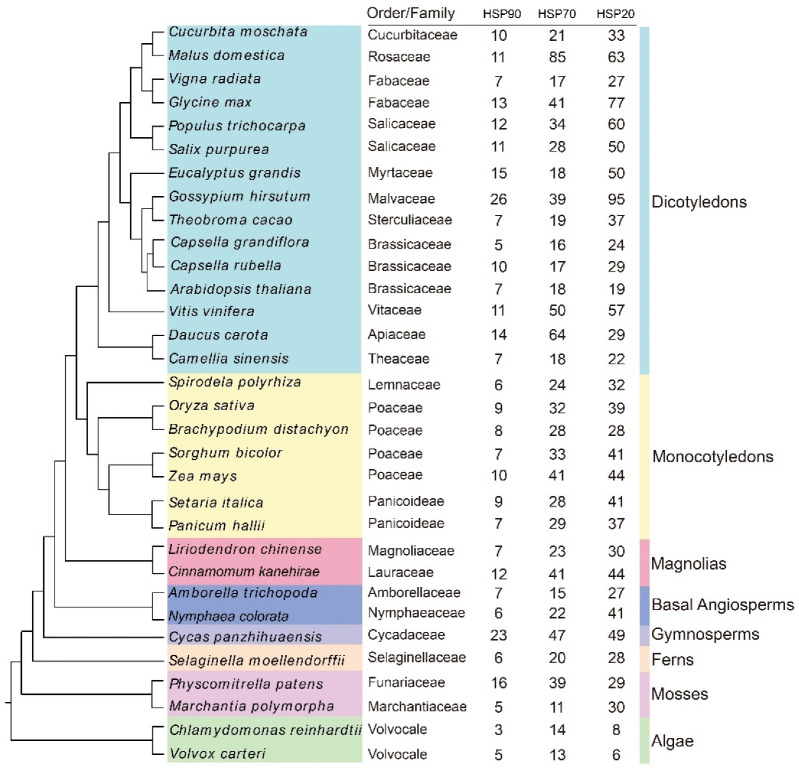
Identification and classification of HSP genes in 32 plant species. The phylogenetic tree was constructed according to the OrthoFinder (v2.5.4). Different color schemes represent different taxons.

To have a deeper understanding of the HSP gene family evolution, we searched the HSP gene family in 32 plants, including 2 algae, 2 mosses, 1 fern, 1 gymnosperm, 2 basal angiosperms, 17 dicotyledons, and 7 monocotyledons using the HMM and BLASTP search (Figure 2, Table S1). We identified a total of 2483 HSP genes (312 HSP90s, 945 HSP70s, and 1226 HSP20s). Among the identified species, *G. hirsutum* and *V. carteri* had the most (160) and the least (24) HSP genes, respectively. Overall, the number of HSPs in the higher plants was higher than that in the lower plants. In contrast, algae and *D. carota* contained the HSP20 family more than the HSP70 and HSP90 family. In the magnolia, the protein sequences of the *C. kanehirae* (97) were greater than those of the *L. chinense* (60), to which we related to the fact that the *C. kanehirae* underwent two whole genome duplication events during evolution as compared to the *L. chinen*s*e*.

### 2.4. Gene Structure and Motif Analysis of the LchiHSP Genes

**Figure 3:**
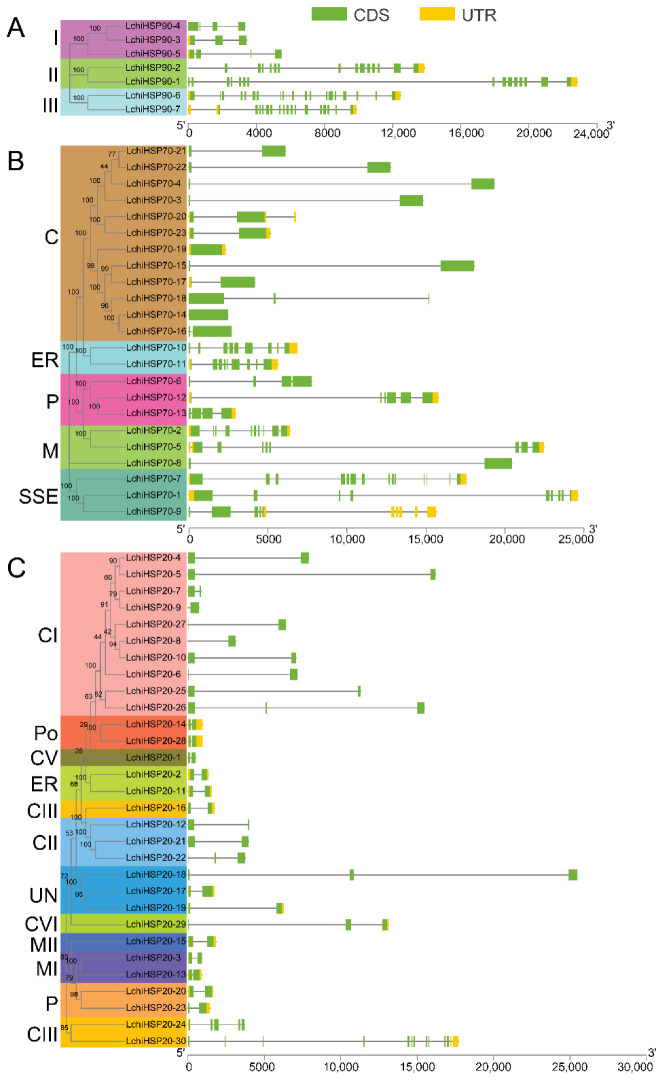
Gene structure analysis of *LchiHSP* genes. (**A**) Gene structure of *LchiHSP90s*. (**B**) Gene structure of *LchiHSP70s*. (**C**) Gene structure of *LchiHSP20s*. C: cytoplasm, CI–CVII: cytoplasm I–VII, ER: endoplasmic reticulum, MI–MII: mitochondria I–II, P: plastid, and Po: peroxide.

Gene structure analysis provides important evidence that supports the evolution of gene families. We investigated the gene structures and motif arrangements of the identified *LchiHSPs* (Figure 3). In the *LchiHSP90* class, different genes showed large differences in the number of introns (2–18) (Figure 3A). The intron number varied greatly among different subfamilies. Group I had 2–3 introns, while groups II and groups III contained 14–18 introns. There were 0–3 introns in the cytoplasmic subfamily of *L. chinense* HSP90 members and 8–13 introns in the SSE subfamily, and two members (*LchiHSP70-14* and *LchiHSP70-19*) contained no intron, and 9 had only 1 intron (Figure 3B). The gene structure of *LchiHSP20s* was relatively conserved compared to *LchiHSP90s* and *LchiHSP70s*. Nonetheless, *LchiHSP20-24* (4) and *LchiHSP20-30* (12) had one to two introns differing from the rest of the *LchiHSP20* members (Figure 3C).

**Figure 4:**
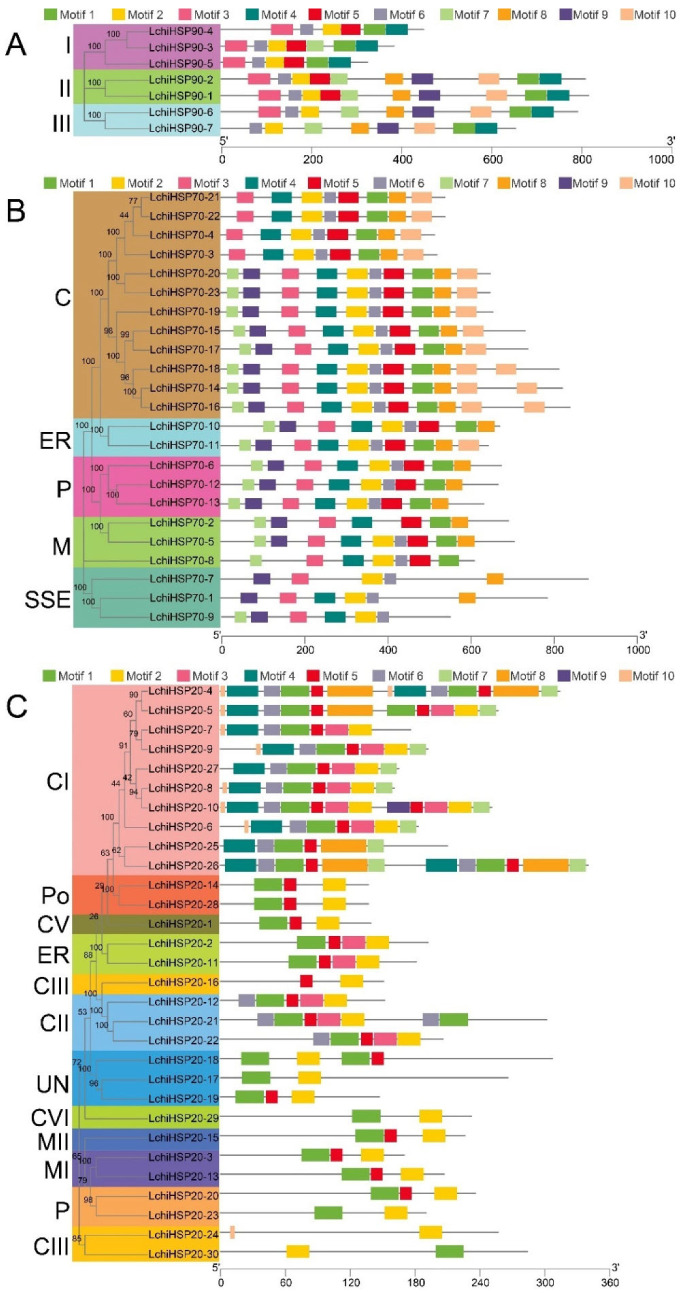
Conserved motifs analysis of LchiHSP genes. (**A**) Conserved motifs of LchiHSP90s. (**B**) Conserved motifs of LchiHSP70s. (**C**) Conserved motifs of LchiHSP20s. C: cytoplasm, CI–CVII: cytoplasm I–VII, ER: endoplasmic reticulum, MI–MII: mitochondria I–II, P: plastid, and Po: peroxide.

We used the online tool, MEME, to identify the conserved motif arrangement in LchiHSPs (Figure 4). The motifs from the same subfamily were identical in number, type, and order. Ten conserved motifs were identified in each LchiHSP superfamily (Table S2). Motif six was assigned as the HATPase_c domain, based on the annotation information from the Pfam database. The other motifs were also shown to belong to the HSP90 conservative domain. In the LchiHSP90 family, motifs 1, 2, 4, and 6 were highly conserved as compared to the other classes (Figure 4A). Protein motif 3 was contained in all 23 LchiHSP70 proteins. The subfamily SSE had the least number of motifs (4–5), with the rest having a motif range between 7 and 10. It is worth mentioning that in the cytoplasm subfamily, the member of LchiHSP all contained 10 motifs except LchiHSP70-3, 4, 21, and 22 (Figure 4B). A further analysis of the 10 conserved motifs revealed that motifs 2, 3, 4, 6, 7, and 9 were the N-terminal ATP binding domains of LchiHSP70s, motifs 1, 8, and 10 were the C-terminal domains, and motif 5 was the subordinate binding domain. In the LchiHSP20 family, motifs 1, 2, and 5 were present in most members. The complete sequences of motifs 1, 2, and 3 together formed a highly conserved whole ACD domain that exhibits vital biological functions (Figure 4C).

### 2.5. Gene Duplication and Collinearity Analysis of the LchiHSP Genes

**Figure 5:**
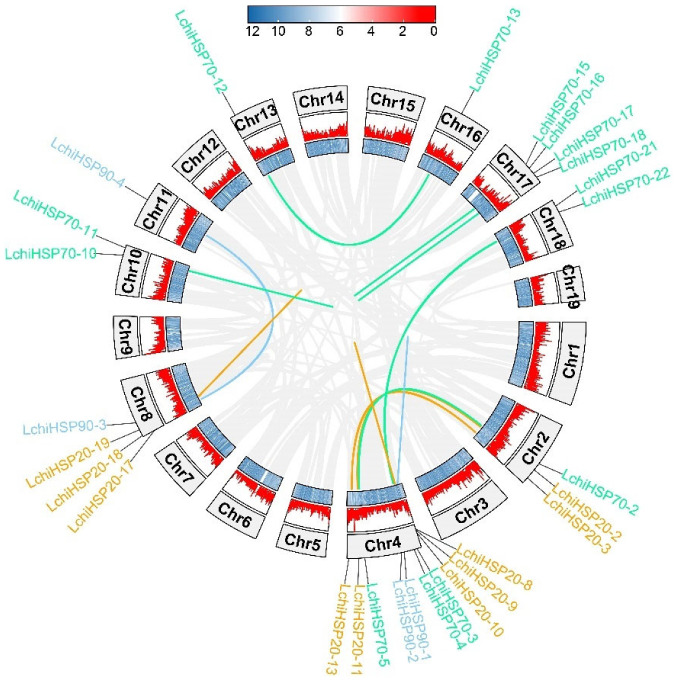
Tandem duplication and segmental duplication of *LchiHSP* genes. The lines of the outer circle and the heatmap represent the gene density on the chromosome, and the gray lines represent all colinear gene pairs of *L. chinense*.

To understand the mechanism of expansion of the *L. chinense HSP* gene family, a genome-wide collinearity analysis was performed. The analysis showed that the *L. chinense HSP* family had undergone a total of 13 gene duplication events, including seven segmental duplication events (*HSP90-3*/*HSP90-4*, *HSP70-12*/*HSP70-13*, *HSP70-3*/*HSP70-21*, *HSP70-4*/*HSP70-22*, *HSP70-2*/*HSP70-5*, *HSP20-2*/*HSP20-11*, *HSP20-3*/*HSP20-13*) and six tandem duplication events (*HSP90-1*/*HSP90-2*, *HSP70-10*/*HSP70-11*, *HSP70-15*/*HSP70-16*, *HSP70-17*/*HSP70-18*, *HSP20-8*/*HSP20-9*/*HSP20-10*, *HSP20-17*/*HSP20-18*/*HSP20-19*) (Figure 5). A further analysis revealed that the duplication events were mainly prevalent on chromosomes 2, 4, 8, and 17, which we termed the important regions for the gene expansion of the *LchiHSP* family.

**Figure 6:**
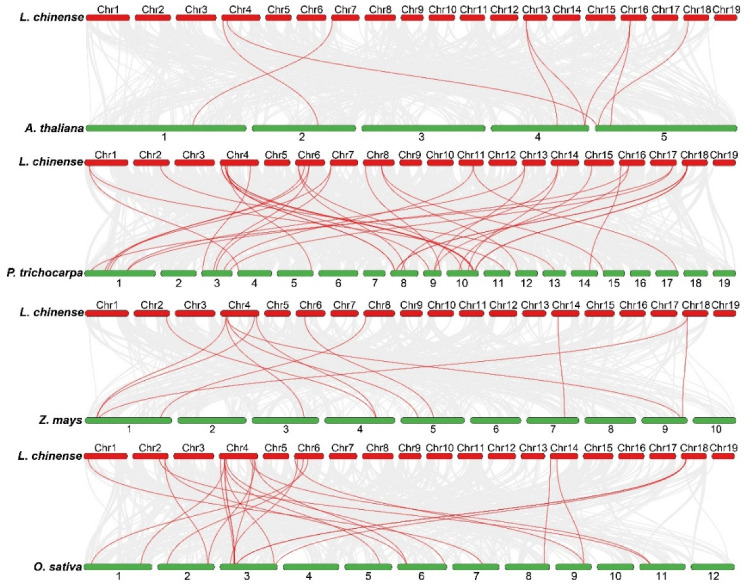
Collinearity analysis between *HSP* genes of *L. chinense* and other species. The red line represents the colinear gene pairs between *LchiHSP* genes and other species, while the grey line represents the colinear gene pairs between the *L. chinense* genome and other species.

The synonymous/non-synonymous (Ka/Ks) analysis of the duplicated *LchiHSP* genes showed a value of less than 1, signifying a purifying selection and also suggesting an evolutionary conservation in the *HSP* gene family of *L. chinense* (Table S3). To further study the evolutionary relationship between the *LchiHSP* family genes and other species *HSPs*, an evolutionary relationship map of the *LchiHSP* genes and other four species was constructed, including 2 dicotyledons (*A. thaliana* and *P. trichocarpa*) and 2 monocotyledons (maize and rice). Dual synteny analysis revealed that there were 8, 37, 12, and 24 orthologs between *L. chinense*/*A. thaliana*, *L. chinense*/*P. trichocarpa*, *L. chinense*/*Z. mays*, and *L. chinense*/*O. sativa*, respectively. It is worth mentioning that *LchiHSP70-4* has orthologous genes in four species (Figure 6, Table S4).

### 2.6. Analysis of Cis-Element in LchiHSP Gene Promoters

**Figure 7:**
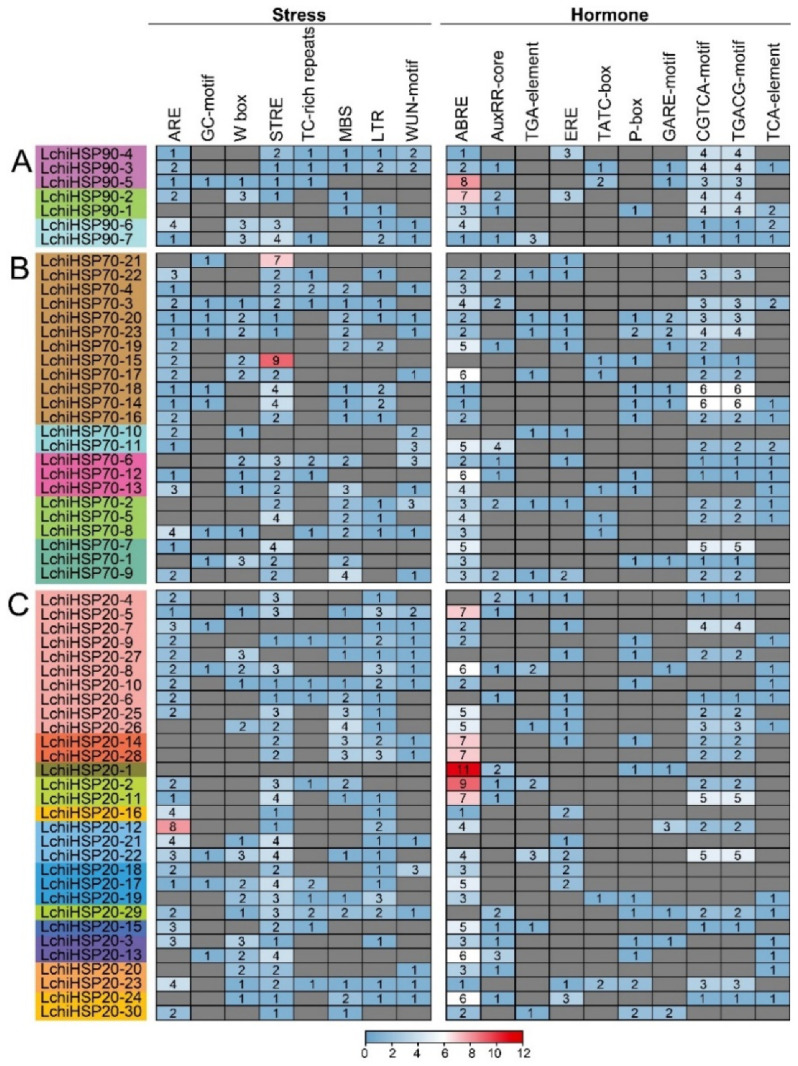
Cis-element analysis of *LchiHSP90* (**A**), *LchiHSP70* (**B**), and *LchiHSP20* (**C**) promoters. It represents the number of various cis-acting elements in the promoter region of each *LchiHSP* gene.

The cis-acting elements in the promoter regions play an important role in the plant’s response to abiotic stress. To gain further insight into the role of the cis-regulatory elements in *LchiHSPs*, we used the PlantCARE website to predict the 2 kb sequence of cis-regulatory elements upstream of *LchiHSPs* (Figure 7 and Figure S2, and Table S5). Here, we focused on hormones and abiotic stress-related cis-acting elements, including MeJA-responsive (CGTCA-motif/TGACG-motif), auxin-responsive (AuxRE-core/TGA-element), ethylene-responsive (ERE), gibberellin-responsive (TATC-box/P-box/GARE-motif), abscisic acid-responsive (ABRE), salicylic acid-responsive (TCA-element), wound responsive (WUN-motif), drought inducibility (MBS), anaerobic inducibility (ARE/GC-motif), low temperature-responsive (LTR), and defense and stress-responsive (W-box/STRE/TC-rich repeats). The analysis showed that the promoters of the *LchiHSP* genes contained different numbers of hormone and abiotic stress-related cis-acting elements. Among them, the ABRE, CGTCA-motif, and TGACG-motif accounted for the largest proportion in the hormone response category, while ARE and STRE accounted for the largest proportion in the stress response category. All the analyzed *LchiHSPs* except for *LchiHSP90-1*, *LchiHSP70-7*, -*11*, and -*21* were shown to carry at least three cis-elements associated with the stress responses, illustrating that *LchiHSPs* are associated with the regulation of abiotic stresses. In addition, *LchiHSP20-22* had the highest number of cis-acting elements (32) in responding to hormones and stress, which leads to the speculation that it may be involved in regulating multiple abiotic stresses (Figure 7).

### 2.7. Three-Dimensional Structures and Protein–Protein Network Analysis of LchiHSP Proteins

**Figure 8:**
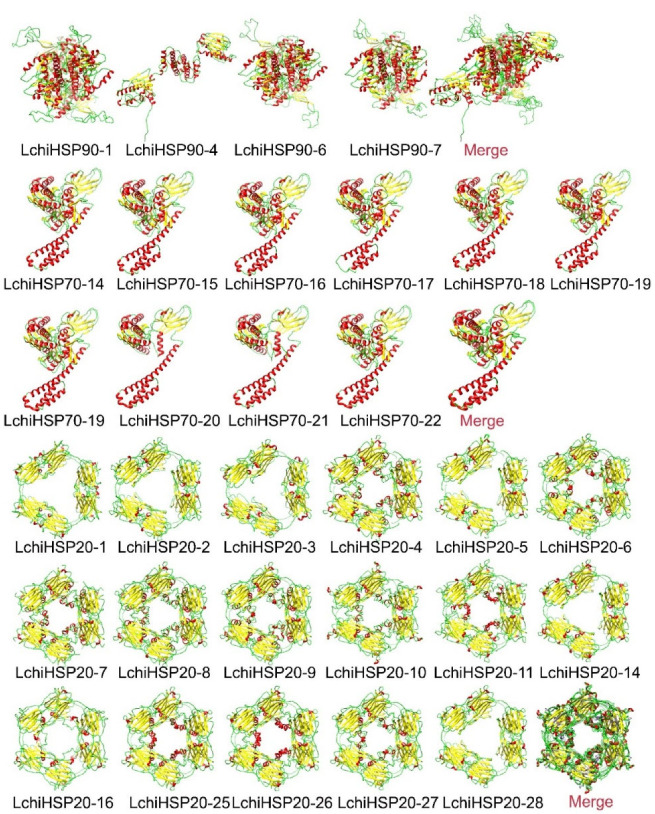
Structural analysis of LchiHSPs. The α-helix, β-strand, and random coil are marked by red, yellow, and green, respectively. The structural images were generated using the Chimera software. The lower right corner of each superfamily is the overlay of all proteins in the same template.

The analysis of the three-dimensional structure of the proteins plays a crucial role in understanding their biological functions, thus we predicted the three-dimensional structure of the identified LchiHSP proteins using SWISS-MODEL homology modeling. We selected 52 LchiHSP models based on a target consistency and template greater than 30% (Figure 8, Table S6). All the proteins were modeled and visualized using the Chimera software based on the template similarity, that is: LchiHSP90 (5uls.1.A), LchiHSP70 (7sqc.391.A), and LchiHSP20 (1gme.2.A). It was found that the structure of the proteins in the same template were identical, except for the fat that LchiHSP90-4 had a large difference compared to the LchiHSP90 family. Additionally, the obtained GMQE values ranged from 0.26 (LchiHSP20-15) to 0.82 (LchiHSP70-23). 

**Figure 9:**
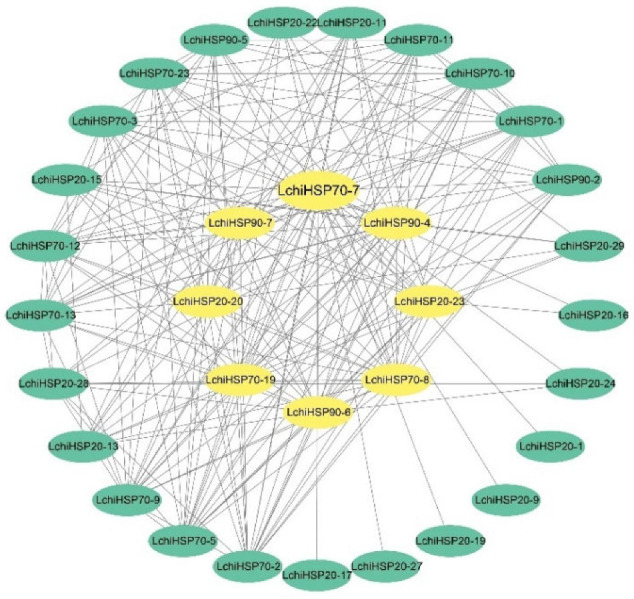
Protein–protein interaction analysis of LchiHSP proteins. Each node represents a protein, and the protein node size indicates the degree of interaction with other nodes.

To further investigate the biological functions of the *LchiHSP* genes, we constructed a protein interaction network using the Online String database, based on the homologous relationship between LchiHSP proteins and *P. trichocarpa*. We noted interactions among 33 LchiHSPs, collectively forming 213 interactions (Table S7). In detail, LchiHSP70-7 (31) had the largest number of interacting proteins HSP, followed by LchiHSP90-4 (21) and LchiHSP90-7 (19), implying that LchiHSP70-7, LchiHSP90-4, and LchiHSP90-7 interact with various protein to fully exhibit their functions (Figure 9).

### 2.8. Expression Analysis of LchiHSPs under Heat and Cold Stress

**Figure 10:**
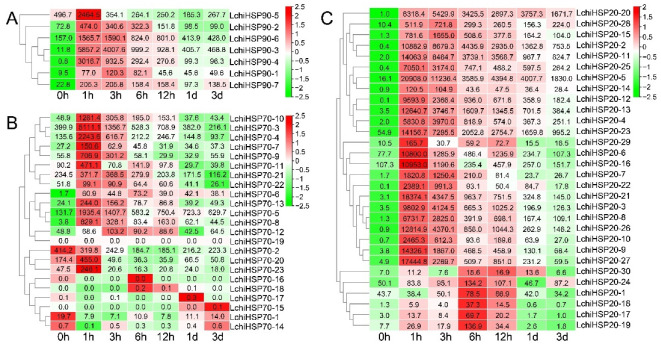
Differential expression of *HSP* genes in *L. chinense* under heat stress. The cluster analysis of *LchiHSPs* expression patterns is shown on the left. Heatmaps of the expression of the: *LchiHSP90* (**A**), *LchiHSP70* (**B**), and *LchiHSP20* (**C**) under heat stress. Different color background indicates differential expression patterns. Numbers show the relative expression value. TPM values of *LchiHSP* genes were log2 transformed.

**Figure 11:**
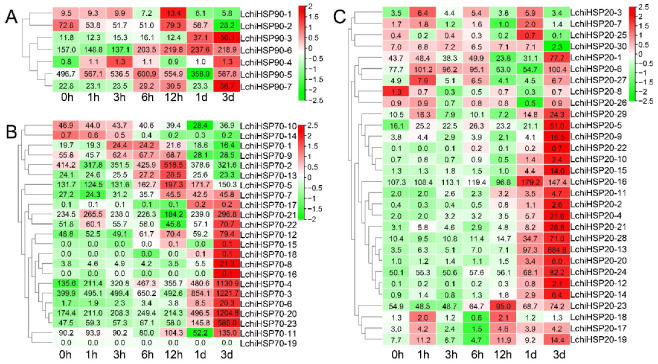
Differential expression of *HSP* genes in *L. chinense* under cold stress. The cluster analysis of *LchiHSPs* expression patterns is shown on the left. Heatmaps of the expression of the *LchiHSP90* (**A**), *LchiHSP70* (**B**), and *LchiHSP20* (**C**) under cold stress. Different color background indicates differential expression. Numbers show relative expression value. TPM values of HSP genes were log2 transformed.

To further understand functions and the expression patterns of the *LchiHSPs* during abiotic stresses, we used the transcriptome data from the leaf tissue under heat and cold stress (Figure 10 and Figure 11, Table S8). Generally, many stress-related genes were differentially expressed at different time points, suggesting the importance of the *HSPHSPs* during abiotic stresses. In detail, all the *LchiHSPSP90* genes were significantly up-regulated under heat stress and reached an expression peak during the first and third hour (Figure 10A). Most of the *LchiHSP70s* were significantly expressed at 1 h of heat stress treatment in varying degrees, whereas *LchiHSP70*-*1*, *-2*, and *-14* were significantly down-regulated (Figure 10B). Furthermore, all the *LchiHSP20s* showed a higher gene expression under a heat stress treatment as compared to *LchiHSP90s* and *LchiHSP70s*. Specifically, six genes (*LchiHSP20*-*1*, *-17*, -*18*, *-19*, -*24*, and *-30*) were expressed the highest after 6 h of the heat treatment (Figure 10C).

In cold stress, the *LchiHSP90* genes were slightly upregulated at different time periods (Figure 11A). In detail, 18 *LchiHSP70s* were responsive to the cold treatment. Specifically, 16 genes had elevated expression patterns while the remaining had not. Nearly 50% of the *LchiHSP70s* genes were expressed the highest at 3 d, indicating that the *LchiHSP70s* are long period cold regulatory genes (Figure 11B). Likewise, most of the *LchiHSP20* genes (18/30) reached their highest expression levels at 3 d. However, during the 0–12 h time period, the expression patterns of *LchiHSP20-3* and *LchiHSP20-6 I* were marked by an expression upregulation initially and a downregulation later, reaching their expression peak at 1 h of cold stress treatment (Figure 11C).

In conclusion, the majority of the *LchiHSP* genes responded to cold and heat stress at different time points and different levels. Based on the transcriptome results, we selected some *LchiHSP* genes for a further validation by qRT-PCR. The results showed that the *LchiHSP90-5*, *LchiHSP70-3,* and *LchiHSP70-4* were induced to different degrees under cold and heat stress. Furthermore, we noted that *LchiHSP20-5* and *LchiHSP20-13* were significantly up-regulated after a heat stress treatment and much lower in a cold stress treatment (Figure S3).

## 3. Discussion

Recent studies have shown that the heat shock proteins are abundantly expressed under stress conditions such as a high temperature, drought, low temperature, and salt stress [24,29,33,34]. They maintain plant homeostasis by participating in various biological functions such as protein synthesis, folding, cellular, and cellular localization, and protein degradation [6,10,17]. In terms of regulation, heat shock proteins are involved in many biological functions, such as protein transmembrane transport and target protein degradation. However, with the continuous progress and wide application of genome sequencing technology, more and more plant HSPs genome data have been published recently, including *Arabidopsis* [19], rice [20], tea plant [35], and *P.trichocarpa* [21]. Nonetheless, the *L. chinense* HSP gene family and its biological functions had not yet been characterized.

In this study, a total of 60 *LchiHSP* genes were identified in the *L. chinense* genome, including 7 *LchiHSP90s*, 23 *LchiHSP70s*, and 30 *LchiHSP20s* (Table 1). The *L. chinense* is considered to be a basal angiosperm [33], suggesting the reason of its small size LchiHSP gene family (Figure 2). It is also possible that the differences in the number of HSP genes are due to differences in the genome size and gene duplication during the plant evolution. Gene duplication events play an important role in the expansion of plant gene families [12,15,16]. The number of HSP genes in the *L. chinense* genome was lower than that in *C. kanehirae* due to the fact that there was only a single WGD event in *L. chinense* and two in *C. kanehirae* [36,37]. Previous studies have demonstrated that the naming of HSPs is based on their subcellular localization and clustering depending phylogenetic relationship [35,38]. In this research, we observed that the classification of the LchiHSPs was to a greater extent consistent with the protein localization, and proteins in the same family were also clustered together by the phylogenetic analysis (Figure 1). Interestingly, we also noted that the M subfamily members were adjacent to the P subfamily members in LchiHSP20s, implying that they may have undergone a tighter differentiation period. Additionally, as there were no LchiHSP20s in the CIV and CVII subfamilies, we related this phenomenon to gene loss during evolution (Figure 1C).

Gene structure plays an important role in the evolution of multigene families, in which the development of introns is an important process in genome evolution and an adaptive measure in species evolution [39]. The lower the number of introns, the greater the ability of the plant to adapt to different developmental processes and environmental stimuli. The analysis of the gene structure of the *LchiHSP90* family members showed differences which we classified into three subfamilies and were consistent with the phylogenetic evolution results (Figure 3A). There was an intronic deletion in *LchiHSP20* genes that has been shown to associate with the induction and rapid processing of proteins after a stress response, and this deletion has also been shown to be responsible for the rapid expression of the *LchiHSP20* genes under various stress conditions (Figure 3C). Gene families and classes are also clustered based on the similarity in the distribution of the conserved motif and research has shown that the proteins in the same group display similar functions. In the research, we noted that the HATPase_c domain composed of motif 1 as the ADP/ATP binding site and had ATPase activity [19,40]. The conserved domain of LchiHSP90, composed of the remaining nine motifs, we speculated to have a key role in maintaining the full movement of the HATPase_c domain. Three highly conserved protein motifs formed the ACD domain [35] and the structural basis for the biological function of LchiHSP20s were detected in most LchiHSP20s, consistent with earlier findings in pepper and switchgrass. We concluded that the diversity of the LchiHSP family is likely driven by environmental selection pressures and the continued evolution of plants (Figure 4).

The cis-regulatory elements are important molecular switches involved in the transcriptional regulation of the gene expression and the control of various biological processes, including the stress responses, hormonal responses, and developmental processes. The cis-regulatory element prediction has been widely used to explore the function of *HSPs* in several species. In this study, various stress-responsive and phytohormone-responsive related cis-elements were found in the promoter region of the *LchiHSPs* (Figure 7), in which the phytohormone-responsive classes accounted for a higher proportion. Phytohormones are a large group of endogenous small, low molecular weight molecules that not only regulate plant growth and development at low concentrations but can also act as signaling molecules involved in plant responses to environmental stresses. Phytohormones such as auxin, ethylene, jasmonic acid, salicylic acid (SA), abscisic acid (ABA), cytokinins (CK), and gibberellins (GAs) play important roles in plant development and stress responses. In higher plants, phytohormones are involved in heat stress signal transduction and regulating the *LchiHSP* expression under heat and cold stress. In addition, the cis-regulatory elements in the promoters are involved in the crosstalk of different stress signals in the gene expression, constituting a gene expression cascade in the abiotic stress response, controlling the molecular processes of the stress response and stress tolerance. Therefore, we observed an interaction between LchiHSPs and phytohormones during environmental stresses, exhibiting important roles in regulating the growth, development, and pressure responses in *L. chinense*. However, future analysis will require further analysis of how the *L. chinense* HSP genes exert their functions. Previous studies have proved that HSP protein interactions regulates plant growth, development, and stress responses [19,40]. HSP90 and HSP70 interact with each other depending on their co-chaperones to form a complex that regulates external stress. In the present study, we also found that interactions between the LchiHSP90 and LchiHSP70 family proteins are ubiquitous and may play an important role in *L. chinense* to high- and low-temperature stress. In addition, some LchiHSP20s were also found to interact with some LchiHSP70s, similar to previous reports on pea and tobacco [41,42]. Therefore, they may act synergistically in stress responses in *L. chinense*.

Gene expression patterns are highly correlated with the function. Therefore, to understand the role of the *LchiHSPs* under heat and cold stress, we selected some genes from each superfamily for a further analysis based on the transcriptome (Figure 10 and Figure 11). The results showed that the expression of all the *LchiHSPs* was significantly increased after the heat stress treatment and gradually decreased with the extension of the treatment period, in which *LchiHSP20s* showed a higher level of gene expression in response to the heat stress treatment compared to the other two superfamilies. Under cold stress, most genes reached the gene expression peak at 3 d, indicating that 3 d is an important time point for cold stress acclimation in *L. chinense*. Furthermore, these gene resources can be provided for plants through gene editing and genetic transformation [43].

## 4. Materials and Methods

### 4.1. Identification and Characterization of HSP Genes in L. chinense

Genome and chromosome annotation information of *L. chinense* were download from the genome database (https://hardwoodgenomics.org/Genome-assembly/2630420, accessed on 15 September 2022), the protein sequences of *Arabidopsis thaliana* HSPs were extracted from TAIR (https://www.arabidopsis.org/, accessed on 15 September 2022) [19,44,45]. Three major heat shock protein family genes were proposed for this study. HSP90, HSP70, and HSP20 family genes were directly extracted from the Pfam database (http://pfam.xfam.org/, accessed on 15 September 2022) using identifier numbers PF00183, PF00012, and PF00011 and applied the E-value < 10^−5^ as the criteria for selection. In addition, the BLASTP search of HSPs using the *A*. *thaliana* HSPs amino acid sequence and the *L*. *chinense* genome database was performed to identify the *L. chinense* HSP gene family members. The NCBI-CDD database (https://www.ncbi.nlm.nih.gov/Structure/cdd/wrpsb.cgi, accessed on 15 September 2022) and SMART website (http://smart.embl-heidelberg.de/, accessed on 15 September 2022) were used to predict the conserved domain of HSPs in *L*. *chinense* [46].

To identify the HSPs in 31 other species, we retrieved genes, proteins, and coding sequences from the Phytozome (https://phytozome-next.jgi.doe.gov/, accessed on 15 September 2022) and NCBI website (https://www.ncbi.nlm.nih.gov/, accessed on 15 September 2022), according to the above-mentioned Hidden Markov Model identification criteria. The 31 species included: algae (*Chlamydomonas reinhardtii* and *Volvox carteri*), mosses (*Marchantia polymorpha* and *Physcomitrella patens*), fern (*Selaginella moellendorffii*), gymnosperm (*Cycas panzhihuaensis*), basal angiosperms (*Nymphaea colorata* and *Amborellatrichopoda*), dicotyledons (*Arabidopsis thaliana*, *Capsella grandiflora*, *Capsella rubella*, *Daucus carota*, *Cucurbita moschata*, *Camellia sinensis*, *Vitis vinifera*, *Salix purpurea*, *Populus trichocarpa*, *Glycine max*, *Vigna radiata*, *Theobroma cacao*, *Malus domestica*, and *Eucalyptus grandis*), magnolia (*Cinnamomum kanehirae*), and monocotyledons (*Spirodela polyrrhiza*, *Brachypodium distachyon*, *Oryza sativa*, *Sorghum bicolor*, *Panicum hallii*, *Zea mays*, and *Setaria italica*).

The protein length, molecular weight (MW), theoretical isoelectric point (PI), instability index, and grand average of the hydropathicity (GRAVY) of HSPs in *L. chinense* were analyzed by the ExPASy Proteomics Server (http://web.expasy.org/protparam, accessed on 15 September 2022). The WoLF PSORT online website (https://wolfpsort.hgc.jp/, accessed on 15 September 2022) predicted the subcellular localization of the *LchiHSP* gene members [47].

### 4.2. Systematic Evolution of HSP Gene Family

To investigate the evolutionary patterns of the HSP gene members in *L*. *chinense*, the *L*. *chinense*, *Arabidopsis*, and rice HSP family protein sequences were collected, and multiple sequence alignments were performed in CLUSTALW (https://www.genome.jp/tools-bin/clustalw, accessed on 15 September 2022). A phylogenetic tree was constructed using MEGA-X software with the neighbor-joining method, and the parameter bootstrap value was set to 1000. The evolutionary tree is beautified through the EvolView website (http://www.evolgenius.info/evolview, accessed on 15 September 2022) [48]. OrthoFinder (v2.5.4) was used to explore the HSP gene evolutionary status of all the 32 species with the FASTA format full-length sequence folder as the parameter ‘-f’ and used the MSA gene tree inference and DIAMOND software to blast quickly. Then, we use MAFFT to perform the multiple sequence alignment, and the IQ-TREE program was used to construct a species tree with the maximum inference likelihood [49].

### 4.3. Gene Structure and Conserved Motif Analysis of LchiHSPs

The exon and intron structure information of the members of the LchiHSPs gene family was obtained from the GFF3 annotation file of the *L*. *chinense* genome, and the conserved protein motifs were analyzed using the online website MEME tool (http://meme-suite.org/tools/meme, accessed on 15 September 2022). The LchiHSPs gene structures and conserved motifs were visualized by the TBtools software [50]; the MEME analysis parameters included a minimum width set to 5 and a full set to 50, the number of conserved motifs was set to 10, and the other parameters were set to the default values.

### 4.4. Chromosome Distribution, Gene Duplication Events, and Collinearity Analysis

The chromosomal location information of all the members of the *L*. *chinense* HSP gene family was downloaded from the *L*. *chinense* genome database. Except for 4 members (*LchiHSP70-23*, *LchiHSP20-28*, *-29*, *-30*) that are not identified on the chromosome, the remaining 56 members can be used to make a chromosome distribution map through the TBtools tool according to their positioning information.

MCscanX was used to study the tandem duplication and segmental duplication events of *LchiHSPs* between different chromosomes of *L*. *chinense*. The Ka/Ks Calculator function in the TBtools software was used to calculate the duplicate gene pairs’ Ka/Ks value. The *L*. *chinense* genome, *Arabidopsis* genome, rice genome, maize genome, and *Populus* genome were analyzed and visualized by TBtools for interspecies collinearity analysis.

### 4.5. Cis-Regulatory Element Analysis of LchiHSP Gene Promoters

The upstream 2000 bp promoter sequences of the *LchiHSPs* were extracted from the *L*. *chinense* genome, and the cis-acting elements were analyzed by the PlantCARE database (http://bioinformatics.psb.ugent.be/webtools/plantcare/html, accessed on 15 September 2022), and the results were visualized by TBtools. The number of cis-acting factors detected is displayed using a heatmap.

### 4.6. Three-Dimensional (3D) Protein Structures Prediction of LchiHSPs

The three-dimensional structure of the LchiHSP proteins determine the specific functions a protein and is essential for understanding the protein functions. The PDB file of the LchiHSP protein was downloaded from the SWISS-MODEL website (https://swissmodel.expasy.org/, accessed on 15 September 2022) based on the homology modelling method according to the sequences of LchiHSP. The PDB files were visualized using Chimera software (http://www.cgl.ucsf.edu/chimera/, accessed on 15 September 2022).

### 4.7. Protein Interaction Network Analysis and Visualization

The protein interaction network of the LchiHSP gene family was predicted in the database STRING (http://string-db.org, accessed on 15 September 2022) based on the protein interaction network information of *P. trichocarpa* and visualized by Cytoscape software (v 3.9.1).

### 4.8. Expression Analysis of LchiHSP Genes

This study cultured 3-month-old somatic embryo seedlings in the greenhouse (16 h day/8 h night, 24 °C, and 70% relative humidity). Heat temperature (40 °C) and low temperature (4 °C) were used to simulate heat and cold stress, respectively. The control group received an equal amount of water in the matrix at 22 °C. Five biological replicates were set per treatment at each time point. After treatment for 1 h, 3 h, 6 h, 12 h, 1 day, and 3 days, respectively, referred to in the previous reports by Li et al. [51], the qRT-PCR primers were designed for selected sequences using the Primer3plus online website (https://www.primer3plus.com/, accessed on 15 September 2022) (Table S9). Using *LchiActin* as the internal reference, each PCR was set to three biological replicates. The relative expression was calculated according to using the 2^–ΔΔCt^ method, and the significance analysis (* *p* < 0.05, ** *p* < 0.01) was performed using Student’s *t*-test in SPSS 26 software, and the graphs of the data were constructed using GraphPad Prism.

## 5. Conclusions

In conclusion, identification and bioinformatics analysis of the LchiHSP genes were performed. The physicochemical properties, comparative phylogeny, gene duplication events, gene structure, cis-acting elements, and protein structure function of LchiHSPs were systematically revealed. The expression patterns of *LchiHSPs* under cold and heat stress were explored, and this study provides a good basis for the subsequent exploration of the functional properties of the *LchiHSPs* gene family members.

## Figures and Tables

**Table 1:** The characteristics of HSP genes in *L. chinense*.

Family	Gene Name	Gene ID	Chr	AA	MW	PI	Instability Index	GRAVY	Subcellular Localization
					(KDa)				
HSP90	LchiHSP90-1	Lchi11721	Chr4	816	93.26	4.94	35.4	−0.68	E.R.
	LchiHSP90-2	Lchi11722	Chr4	809	92.36	4.91	32.9	−0.73	nucl
	LchiHSP90-3	Lchi28127	Chr8	384	43.31	4.5	40.7	−0.49	cyto
	LchiHSP90-4	Lchi13166	Chr11	450	50.66	4.98	33.5	−0.29	cyto
	LchiHSP90-5	Lchi19077	Chr14	325	36.14	4.46	33.1	−0.32	plas
	LchiHSP90-6	Lchi20964	Chr14	792	90.22	4.99	49.9	−0.49	chlo
	LchiHSP90-7	Lchi07189	Chr16	654	74.29	5.06	34.7	−0.59	chlo
HSP70	LchiHSP70-1	Lchi21749	Chr1	786	86.84	5.23	47.0	−0.33	cyto
	LchiHSP70-2	Lchi01584	Chr2	694	74.45	5.52	35.4	−0.25	chlo
	LchiHSP70-3	Lchi18956	Chr4	520	57.22	5.31	34.0	−0.44	chlo
	LchiHSP70-4	Lchi18963	Chr4	514	56.63	5.06	37.6	−0.49	cyto
	LchiHSP70-5	Lchi16202	Chr4	708	75.26	5.49	31.5	−0.27	chlo
	LchiHSP70-6	Lchi06739	Chr6	676	73.17	5.31	35.9	−0.17	E.R.
	LchiHSP70-7	Lchi06856	Chr6	884	98.26	5.42	37.7	−0.41	E.R.
	LchiHSP70-8	Lchi12038	Chr6	612	66.35	5.13	39.3	0.04	cyto
	LchiHSP70-9	Lchi17471	Chr6	550	60.67	7.88	45.4	−0.06	cyto
	LchiHSP70-10	Lchi28754	Chr10	671	74.65	8.9	31.1	−0.40	chlo
	LchiHSP70-11	Lchi28755	Chr10	644	71.43	5.12	29.7	−0.51	E.R.
	LchiHSP70-12	Lchi18241	Chr13	669	71.67	5.79	37.6	−0.31	mito
	LchiHSP70-13	Lchi17994	Chr16	634	68.03	5.33	38.6	−0.30	cyto
	LchiHSP70-14	Lchi28433	Chr17	822	88.99	4.79	35.8	−0.37	cyto
	LchiHSP70-15	Lchi29184	Chr17	732	80.11	5.2	33.6	−0.41	cyto
	LchiHSP70-16	Lchi29183	Chr17	840	91.01	4.74	38.5	−0.41	cyto
	LchiHSP70-17	Lchi29180	Chr17	739	80.76	5.26	33.3	−0.34	mito
	LchiHSP70-18	Lchi29179	Chr17	813	88.72	5.05	33.3	−0.28	cyto
	LchiHSP70-19	Lchi03740	Chr17	655	71.64	5.19	35.3	−0.38	cyto
	LchiHSP70-20	Lchi21178	Chr18	648	71.05	5.09	34.0	−0.43	cyto
	LchiHSP70-21	Lchi21185	Chr18	539	59.06	5.32	34.6	−0.46	cyto
	LchiHSP70-22	Lchi24313	Chr18	539	59.64	5.13	36.9	−0.51	cyto
	LchiHSP70-23	Lchi34042	Scaffold674	648	71.05	5.09	34.0	−0.43	cyto
HSP20	LchiHSP20-1	Lchi21756	Chr1	139	16.13	5.2	48.3	−0.30	cyto
	LchiHSP20-2	Lchi01684	Chr2	192	21.97	5.78	32.5	−0.40	vacu
	LchiHSP20-3	Lchi13753	Chr2	170	19.11	6.77	63.8	−0.70	mito
	LchiHSP20-4	Lchi04244	Chr4	314	36.03	6.23	59.1	−0.66	cyto
	LchiHSP20-5	Lchi04241	Chr4	257	29.61	7.86	51.4	−0.76	cyto
	LchiHSP20-6	Lchi04239	Chr4	183	20.27	6.19	54.3	−0.45	cyto
	LchiHSP20-7	Lchi04235	Chr4	176	20.12	5.4	45.6	−0.68	cyto
	LchiHSP20-8	Lchi04233	Chr4	161	18.50	6.78	44.4	−0.70	cyto
	LchiHSP20-9	Lchi04232	Chr4	192	21.80	9.2	53.6	−0.62	cyto
	LchiHSP20-10	Lchi04231	Chr4	251	28.97	9.24	52.9	−0.78	cyto
	LchiHSP20-11	Lchi01989	Chr4	181	20.53	6.87	46.1	−0.49	extr
	LchiHSP20-12	Lchi02197	Chr4	152	17.13	5.08	41.0	−0.71	chlo
	LchiHSP20-13	Lchi09721	Chr4	207	23.46	5.49	66.8	−0.61	mito
	LchiHSP20-14	Lchi25013	Chr5	137	15.57	6.85	37.8	−0.39	pero
	LchiHSP20-15	Lchi05522	Chr6	226	25.99	9.01	52.8	−0.75	mito
	LchiHSP20-16	Lchi11461	Chr7	151	16.73	6.1	52.4	−0.42	cyto
	LchiHSP20-17	Lchi20267	Chr8	266	29.12	9.34	37.9	−0.65	cyto
	LchiHSP20-18	Lchi20268	Chr8	307	35.25	9.15	35.6	−0.59	chlo
	LchiHSP20-19	Lchi20269	Chr8	147	16.88	9.3	43.5	−0.66	golg
	LchiHSP20-20	Lchi06582	Chr8	236	26.51	8.24	53.0	−0.55	chlo
	LchiHSP20-21	Lchi24852	Chr11	302	34.14	4.96	45.6	−0.77	cyto
	LchiHSP20-22	Lchi24857	Chr11	206	23.64	6.87	53.1	−0.43	chlo
	LchiHSP20-23	Lchi19505	Chr15	190	21.49	9	44.8	−0.81	nucl
	LchiHSP20-24	Lchi03985	Chr15	257	28.99	6.32	58.9	−0.49	cyto
	LchiHSP20-25	Lchi03949	Chr15	210	23.85	9.57	57.4	−0.53	cyto
	LchiHSP20-26	Lchi03941	Chr15	340	38.71	6.45	58.3	−0.63	chlo
	LchiHSP20-27	Lchi28031	Chr18	165	18.87	6.21	43.0	−0.66	cyto
	LchiHSP20-28	Lchi31103	Scaffold97	137	15.59	6.85	37.8	−0.39	pero
	LchiHSP20-29	Lchi26427	Scaffold941	232	26.84	5.06	65.9	−0.38	chlo
	LchiHSP20-30	Lchi31495	Scaffold1647	284	31.74	9.16	42.5	−0.40	cyto

Chlo: chloroplast; cyto: cytoplasm; ER: endoplasmic reticulum; golg: golgi apparatus; mito: mitochondria; nucl: nucleus; pero: peroxide; plas: plasma membrane; vacu: vacuole.

## Data Availability

Not applicable.

## Supplementary Materials

The following supporting information can be downloaded at: https://www.mdpi.com/article/10.3390/ijms232315051/s1.

## References

[1] Kidokoro S., Shinozaki K., Yamaguchi-Shinozaki K. (2022). Transcriptional regulatory network of plant cold-stress responses. Trends Plant Sci..

[2] Schulz P., Piepenburg K., Lintermann R., Herde M., Schottler M.A., Schmidt L.K., Ruf S., Kudla J., Romeis T., Bock R. (2021). Improving plant drought tolerance and growth under water limitation through combinatorial engineering of signalling networks. Plant Biotechnol. J..

[3] Shang J.X., Li X.Y., Li C.L., Zhao L.Q. (2022). The role of nitric oxide in plant responses to salt stress. Int. J. Mol. Sci..

[4] Gill R.A., Ahmar S., Ali B., Saleem M.H., Khan M.U., Zhou W., Liu S. (2021). The role of membrane transporters in plant growth and development, and abiotic stress tolerance. Int. J. Mol. Sci..

[5] Waadt R., Seller C.A., Hsu P.K., Takahashi Y., Munemasa S., Schroeder J.I. (2022). Plant hormone regulation of abiotic stress responses. Nat. Rev. Mol. Cell Biol..

[6] Wang W., Vinocur B., Shoseyov O., Altman A. (2004). Role of plant heat-shock proteins and molecular chaperones in the abiotic stress response. Trends Plant Sci..

[7] Lindquist S. (1986). The heat-shock response. Annu. Rev. Biochem..

[8] Sun X., Huang N., Li X., Zhu J., Bian X., Li H., Wang L., Hu Q., Luo H. (2021). A chloroplast heat shock protein modulates growth and abiotic stress response in creeping bentgrass. Plant Cell Environ..

[9] Lindquist S., Craig E.A. (1988). The heat-shock proteins. Annu. Rev. Genet..

[10] Driedonks N., Xu J., Peters J.L., Park S., Rieu I. (2015). Multi-level interactions between heat shock factors, heat shock proteins, and the redox system regulate acclimation to heat. Front. Plant Sci..

[11] Wang X., Zheng Y., Chen B., Zhi C., Qiao L., Liu C., Pan Y., Cheng Z. (2021). Genome-wide identification of small heat shock protein (HSP20) homologs in three cucurbit species and the expression profiles of CsHSP20s under several abiotic stresses. Int. J. Biol. Macromol..

[12] Tabusam J., Shi Q., Feng D., Zulfiqar S., Shen S., Ma W., Zhao J. (2022). HSP70 gene family in *Brassica rapa*: Genome-wide identification, characterization, and expression patterns in response to heat and cold stress. Cells.

[13] Yu H., Yang Z., Sui M., Cui C., Hu Y., Hou X., Xing Q., Huang X., Bao Z. (2021). Identification and characterization of HSP90 gene family reveals involvement of HSP90, GRP94 and not TRAP1 in heat stress response in *Chlamys farreri*. Genes.

[14] Song Z., Pan F., Yang C., Jia H., Jiang H., He F., Li N., Lu X., Zhang H. (2019). Genome-wide identification and expression analysis of HSP90 gene family in *Nicotiana tabacum*. BMC Genet..

[15] Davoudi M., Chen J., Lou Q. (2022). Genome-wide identification and expression analysis of heat shock protein 70 (HSP70) gene family in pumpkin (*Cucurbita moschata*) rootstock under drought stress suggested the potential role of these chaperones in stress tolerance. Int. J. Mol. Sci..

[16] Rehman A., Atif R.M., Qayyum A., Du X., Hinze L., Azhar M.T. (2020). Genome-wide identification and characterization of HSP70 gene family in four species of cotton. Genomics.

[17] Waters E.R., Vierling E. (2020). Plant small heat shock proteins—Evolutionary and functional diversity. New Phytol..

[18] Gao T., Mo Z., Tang L., Yu X., Du G., Mao Y. (2022). Heat shock protein 20 gene superfamilies in red algae: Evolutionary and functional diversities. Front. Plant Sci..

[19] Krishna P., Gloor G. (2001). The Hsp90 family of proteins in *Arabidopsis thaliana*. Cell Stress Chaperones.

[20] Jung K.H., Gho H.J., Nguyen M.X., Kim S.R., An G. (2013). Genome-wide expression analysis of HSP70 family genes in rice and identification of a cytosolic HSP70 gene highly induced under heat stress. Funct. Integr. Genom..

[21] Zhang J., Liu B., Li J., Zhang L., Wang Y., Zheng H., Lu M., Chen J. (2015). Hsf and Hsp gene families in *Populus*: Genome-wide identification, organization and correlated expression during development and in stress responses. BMC Genom..

[22] Jiang L., Hu W., Qian Y., Ren Q., Zhang J. (2021). Genome-wide identification, classification and expression analysis of the Hsf and Hsp70 gene families in maize. Gene.

[23] Sun W., Bernard C., van de Cotte B., Van Montagu M., Verbruggen N. (2001). At-HSP17.6A, encoding a small heat-shock protein in *Arabidopsis*, can enhance osmotolerance upon overexpression. Plant J..

[24] Jung Y.J., Nou I.S., Kang K.K. (2014). Overexpression of *Oshsp16.9* gene encoding small heat shock protein enhances tolerance to abiotic stresses in rice. Plant Breed. Biotech..

[25] Guo M., Liu J.H., Ma X., Zhai Y.F., Gong Z.H., Lu M.H. (2016). Genome-wide analysis of the Hsp70 family genes in pepper (*Capsicum annuum* L.) and functional identification of CaHsp70-2 involvement in heat stress. Plant Sci..

[26] Yogendra K.N., Kumar A., Sarkar K., Li Y., Pushpa D., Mosa K.A., Duggavathi R., Kushalappa A.C. (2015). Transcription factor StWRKY1 regulates phenylpropanoid metabolites conferring late blight resistance in potato. J. Exp. Bot..

[27] Kallamadi P.R., Dandu K., Kirti P.B., Rao C.M., Thakur S.S., Mulpuri S. (2018). An insight into powdery mildew-infected, susceptible, resistant, and immune sunflower genotypes. Proteomics.

[28] Farooq M.A., Zhang X., Zafar M.M., Ma W., Zhao J. (2021). Roles of reactive oxygen species and mitochondria in seed germination. Front. Plant Sci..

[29] Ashraf M., Mao Q., Hong J., Shi L., Ran X., Liaquat F., Uzair M., Liang W., Fernie A.R., Shi J. (2021). HSP70-16 and VDAC3 jointly inhibit seed germination under cold stress in *Arabidopsis*. Plant Cell Environ..

[30] Samakovli D., Roka L., Dimopoulou A., Plitsi P.K., Zukauskait A., Georgopoulou P., Novak O., Milioni D., Hatzopoulos P. (2021). HSP90 affects root growth in *Arabidopsis* by regulating the polar distribution of PIN1. New Phytol..

[31] Cao Y., Feng J., Hwarari D., Ahmad B., Wu H., Chen J., Yang L. (2022). Alterations in population distribution of *Liriodendron chinense* (Hemsl.) Sarg. and *Liriodendron tulipifera* Linn. caused by climate change. Forests.

[32] Tu Z., Shen Y., Wen S., Zong Y., Li H. (2020). Alternative splicing enhances the transcriptome complexity of *Liriodendron chinense*. Front. Plant Sci..

[33] Wang J., Chen L., Long Y., Si W., Cheng B., Jiang H. (2021). A novel heat shock transcription factor (*ZmHsf08*) negatively regulates salt and drought stress responses in Maize. Int. J. Mol. Sci..

[34] Zhao Y., Du H., Wang Y., Wang H., Yang S., Li C., Chen N., Yang H., Zhang Y., Zhu Y. (2021). The calcium-dependent protein kinase ZmCDPK7 functions in heat-stress tolerance in maize. J. Integr. Plant Biol..

[35] Chen J., Gao T., Wan S., Zhang Y., Yang J., Yu Y., Wang W. (2018). Genome-wide identification, classification and expression analysis of the HSP gene superfamily in tea plant (*Camellia sinensis*). Int. J. Mol. Sci..

[36] Chen J., Hao Z., Guang X., Zhao C., Wang P., Xue L., Zhu Q., Yang L., Sheng Y., Zhou Y. (2019). Liriodendron genome sheds light on angiosperm phylogeny and species-pair differentiation. Nat. Plants.

[37] Chaw S.M., Liu Y.C., Wu Y.W., Wang H.Y., Lin C.I., Wu C.S., Ke H.M., Chang L.Y., Hsu C.Y., Yang H.T. (2019). Stout camphor tree genome fills gaps in understanding of flowering plant genome evolution. Nat. Plants.

[38] Yao F., Song C., Wang H., Song S., Jiao J., Wang M., Zheng X., Bai T. (2020). Genome-wide characterization of the *HSP20* gene family identifies potential members involved in temperature stress response in apple. Front Genet..

[39] Zhao P., Wang D., Wang R., Kong N., Zhang C., Yang C., Wu W., Ma H., Chen Q. (2018). Genome-wide analysis of the potato *Hsp20* gene family: Identification, genomic organization and expression profiles in response to heat stress. BMC Genom..

[40] Wang L., Liu F., Ju L., Xue B., Wang Y., Wang D., Hou D. (2022). Genome structures and evolution analysis of *Hsp90* gene family in *Brassica napus* reveal the possible roles of members in response to salt stress and the infection of *Sclerotinia sclerotiorum*. Front. Plant Sci..

[41] Lee G.J., Vierling E. (2000). A small heat shock protein cooperates with heat shock protein 70 systems to reactivate a heat-denatured protein. Plant Physiol..

[42] Mogk A., Deuerling E., Vorderwülbecke S., Vierling E., Bukau B. (2003). Small heat shock proteins, ClpB and the DnaK system form a functional triade in reversing protein aggregation. Mol. Microbiol..

[43] Min T., Hwarari D., Li D., Movahedi A., Yang L. (2022). CRISPR-based genome editing and its applications in woody plants. Int. J. Mol. Sci..

[44] Lin B.L., Wang J.S., Liu H.C., Chen R.W., Meyer Y., Barakat A., Delseny M. (2001). Genomic analysis of the Hsp70 superfamily in *Arabidopsis thaliana*. Cell Stress Chaperones.

[45] Scharf K.D., Siddique M., Vierling E. (2001). The expanding family of *Arabidopsis thaliana* small heat stress proteins and a new family of proteins containing alpha-crystallin domains (Acd proteins). Cell Stress Chaperones.

[46] Hwarari D., Guan Y., Li R., Movahedi A., Chen J., Yang L. (2022). Comprehensive bioinformatics and expression analysis of TCP transcription factors in *Liriodendron chinense* reveals putative abiotic stress regulatory roles. Forests.

[47] Ke Y., Xu M., Hwarari D., Ahmad B., Li R., Guan Y., Chen J., Yang L. (2022). OSCA Genes in *Liriodendron chinense*: Characterization, evolution and response to abiotic stress. Forests.

[48] Li R., Radani Y., Ahmad B., Movahedi A., Yang L. (2022). Identification and characteristics of SnRK genes and cold stress-induced expression profiles in *Liriodendron chinense*. BMC Genom..

[49] Wu W., Zhu S., Xu L., Zhu L., Wang D., Liu Y., Liu S., Hao Z., Lu Y., Yang L. (2022). Genome-wide identification of the *Liriodendron chinense* WRKY gene family and its diverse roles in response to multiple abiotic stress. BMC Plant Biol..

[50] Li M., Hwarari D., Li Y., Ahmad B., Min T., Zhang W., Wang J., Yang L. (2022). The bZIP transcription factors in *Liriodendron chinense*: Genome-wide recognition, characteristics and cold stress response. Front. Plant Sci..

[51] Li R., Ahmad B., Hwarari D., Li D., Lu Y., Gao M., Chen J., Yang L. (2022). Genomic survey and cold-induced expression patterns of bHLH transcription factors in *Liriodendron chinense* (Hemsl) Sarg. Forests.

