# Supplementary material for: Genomic Survey of Heat Shock Proteins in Liriodendron chinense Provides Insight into Evolution, Characterization, and Functional Diversities
Source: Int J Mol Sci. 2022 Nov 30;23(23):15051. doi: 10.3390/ijms232315051 (PMC9739435; doi:10.3390/ijms232315051)
Supplement: Supplementary file 1 [file ijms-23-15051-s001.zip › ijms-1989143 - supplementary figures.pdf]

International Journal of *Molecular Sciences*

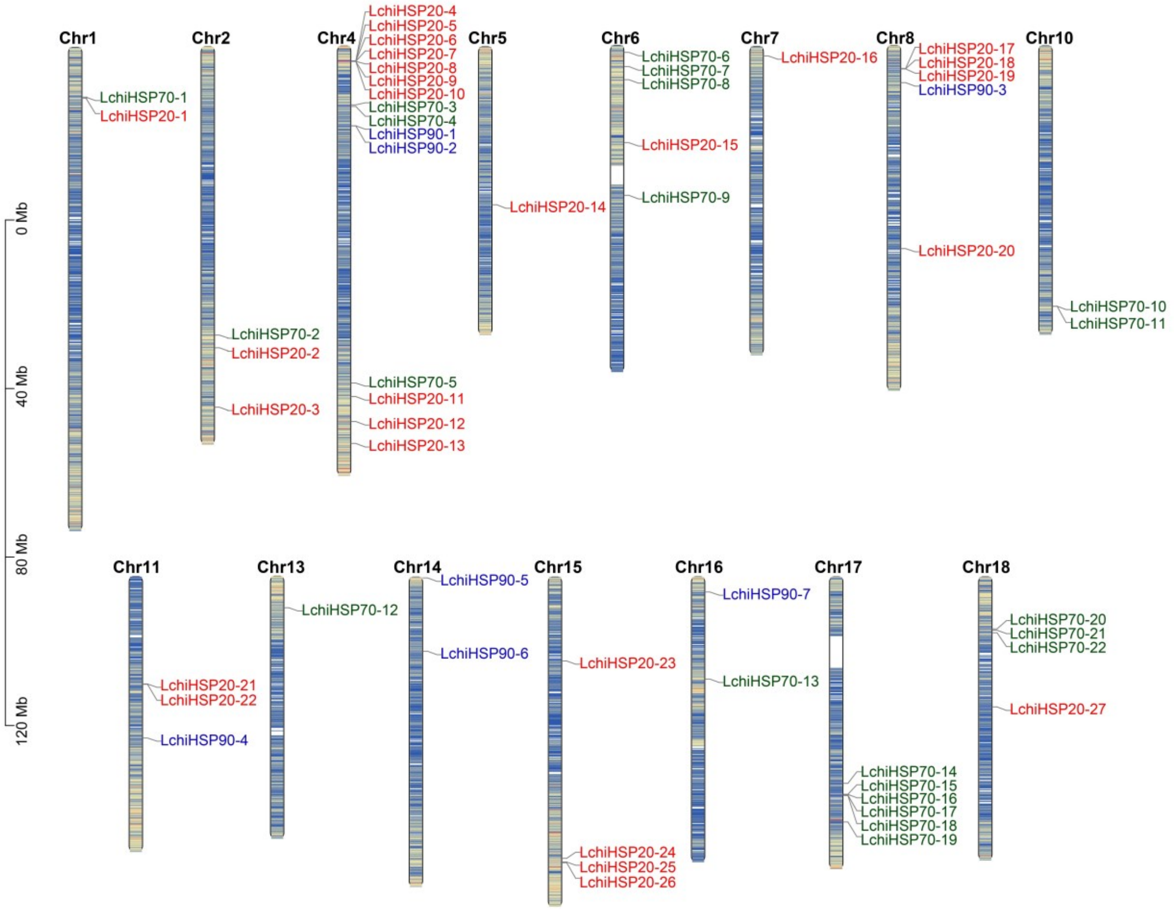

Figure S1. Chromosome distribution of HSP members in *L. chinense*.

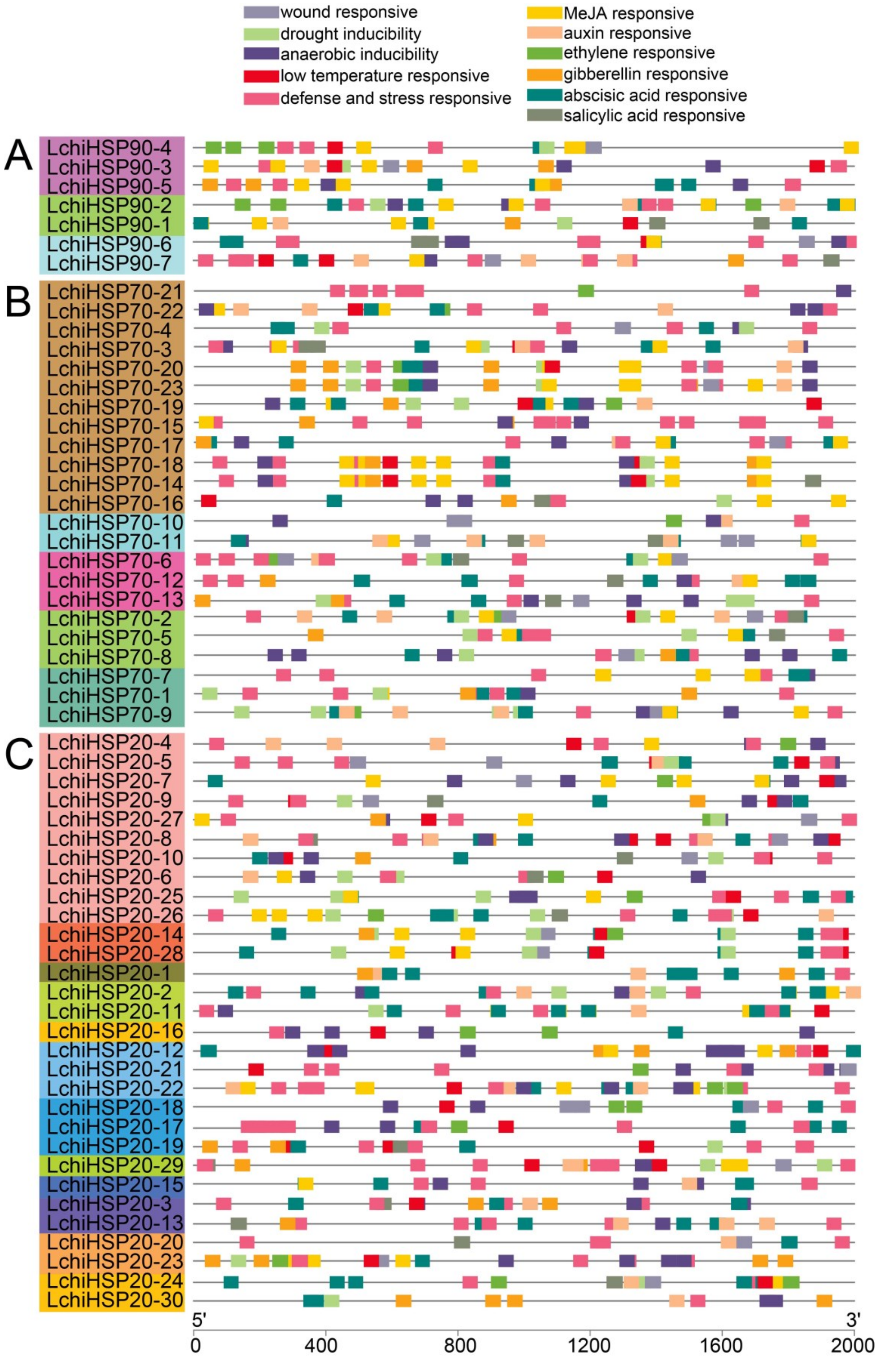

Figure S2. *LchiHSP90* (**A**), *LchiHSP70* (**B**), and *LchiHSP20* (**C**). Squares with different colors on the right represent different types of *LchiHSPs* cis-acting elements and their positions in the promoter region.

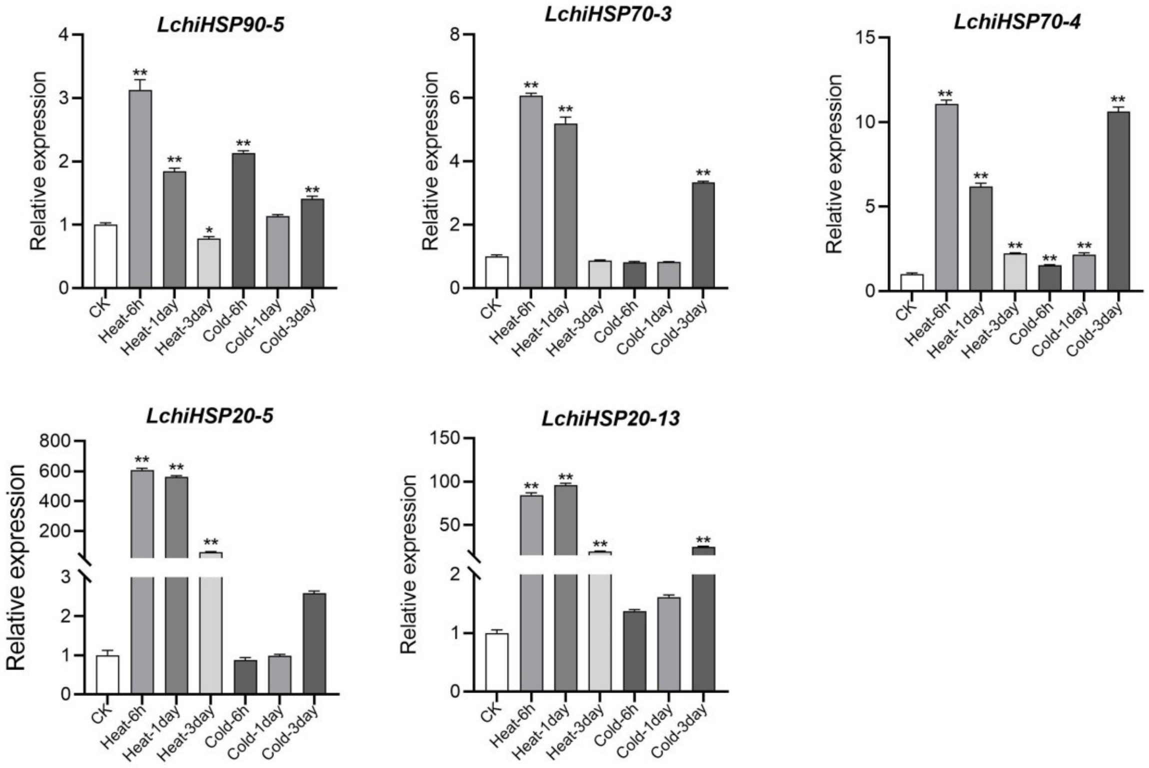

Figure S3. Expression analysis of *LchiHSPs* genes in response to cold and heat stress. * $p < 0.05$, ** $p < 0.01$.
